# Portal Venous Thrombosis Associated with Use of Etonogestrel/ethinyl Estradiol Vaginal Ring

**DOI:** 10.5811/cpcem.2020.1.44654

**Published:** 2020-04-14

**Authors:** Katelynn E. Bailey, Michael J. Tranovich

**Affiliations:** * Charleston Area Medical Center, Department of Emergency Medicine, Charleston, West Virginia; † Allegheny Health Network, Department of Emergency Medicine, Canonsburg, Pennsylvania

**Keywords:** portal venous thrombosis, etonogestrel/ethinyl vaginal ring, contraception, complications

## Abstract

**Introduction:**

Portal venous thrombosis is a life-threatening cause of abdominal pain. In younger patients, heritable thrombophilias, pregnancy, tobacco use, and oral contraceptives are associated.

**Case Report:**

A 26-year-old woman prescribed contraceptive vaginal ring presented with abdominal pain and was diagnosed with an extensive portal venous thrombosis. Management included heparin and later an oral anticoagulant with good short-term outcome.

**Discussion:**

Women using hormonal contraception are approximately four times more likely to develop thromboembolism. Risk of thromboembolism is similar between users of intravaginal and oral contraceptives.

**Conclusion:**

Portal venous thrombosis must be considered in women presenting with abdominal pain who are prescribed hormonal contraceptives, including intravaginal forms.

## INTRODUCTION

Abdominal pain continues to be one of the most common complaints evaluated in the emergency department (ED) in the United States. According to Meltzer et al abdominal pain comprised approximately 23 million visits to the ED in one year.^1^ Comparatively, mesenteric venous thrombosis is a relatively rare condition, accounting for less than 0.02% of all hospital admissions.^2^,^3^ The mean age of mesenteric thrombosis is 45–60 years, with a large proportion occurring in the sixth and seventh decades.^2^–^4^ In younger individuals, mesenteric thrombosis is often associated with the following: heritable thrombophilias (e.g., protein C or protein S deficiency); acquired thrombophilias (e.g., pregnancy or medication use, commonly oral contraceptives); intra-abdominal causes (e.g., cirrhosis or trauma); or idiopathic causes.^2^ This is a case of a 26-year-old woman who presented to the ED with right upper quadrant abdominal pain due to extensive thrombus within the superior mesenteric, main portal, distal splenic, and intrahepatic portal veins. After extensive diagnostic testing, the only identified thrombotic risk factor was the use of an etonogestrel/ethinyl estradiol vaginal ring (NuvaRing).

## CASE REPORT

A 26-year-old woman without medical history presented to the ED due to abdominal pain for approximately 12 hours. The patient reported no tobacco use, and her only prescribed medication was the etonogestrel/ethinyl estradiol intravaginal ring. The patient had been evaluated earlier in the day at an urgent care facility; the urgent care provider then sent the patient to the ED for an apparent abnormal urinalysis (UA). In the ED, she was complaining of mid-epigastric and right upper quadrant abdominal pain along with continued nausea. She also noted back pain, a headache, and bilateral upper extremity numbness since that morning. She noted her bilateral upper extremity numbness and headache had become intermittent. She denied any associated diarrhea, constipation, dysuria, fever, chills, recent travel, or trauma.

The patient’s vital signs included the following: temperature 98.0º Fehrenheit; heart rate 76 beats per minute; respirations 18 breaths per minute; blood pressure 129/53 millimeters of mercury; and pulse oximetry 98% on room air. On physical exam, she exhibited moderate tenderness to palpation of the right upper quadrant and epigastric area. The remainder of the physical exam was unremarkable.

There were no considerable lab abnormalities except for “small” bilirubin noted on the UA. Urine pregnancy test was negative. The patient had a computed tomography (CT) of the abdomen/pelvis with intravenous (IV) contrast, which demonstrated an extensive thrombus within the superior mesenteric vein, extending into the main portal vein, intrahepatic portal veins, and distal splenic vein (Image 1). She was administered IV heparin 5800 units bolus and a continuous IV heparin infusion of 18 units per kilogram per hour. She was then transferred to a tertiary care center.

At the tertiary care center, the patient was maintained on the heparin infusion until she was later transitioned to rivaroxaban. The patient underwent extensive hematologic testing including the following: protein C; protein S; anti-thrombin III; alpha fetoprotein, homocysteine; factor 5 gene mutation; prothrombin gene mutation; anti-cardiolipin antibody IgG and IgM by ELISA; anti-beta2-GP I antibody; JAK2 V617F mutation; and mutation in exon 12 of JAK2. No abnormalities were detected. The patient also had normal venous Doppler studies of the bilateral upper and lower extremities. Ultimately she was discharged home on hospital day 3 on rivaroxaban 15 milligrams twice daily with hematology follow-up and discontinuation of hormonal contraception.

**CPC-EM Capsule**

What do we already know about this clinical entity?

Portal venous thrombosis is well studied in the literature and has known risk factors such as age, heritable and acquired coagulopathies, and idiopathic causes.

What makes this presentation of disease reportable?

This patient’s only risk factor predisposing her to a large portal venous thrombosis was the use of an etonogestrel/ ethinyl estradiol vaginal ring contraceptive.

What is the major learning point?

Emergency physicians should be cognizant that all forms of contraception with exogenous hormones expose patients to a risk of significant thrombosis.

How might this improve emergency medicine practice?

Practitioner awareness regarding thrombotic risk related to all forms of hormonal contraception, including intravaginal forms, may assist in accurate diagnosis.

## DISCUSSION

The clinical presentation, diagnosis, and management of portal and mesenteric venous thrombosis has been thoroughly investigated in the literature. In the acute setting, mesenteric venous thrombosis most commonly causes abdominal pain in 91–100% of cases.^5^ The test of choice for the diagnosis of mesenteric venous thrombosis is a contrast-enhanced CT.^6^ Laboratory testing is not especially helpful as most tests do not correlate specifically to the diagnosis of mesenteric thrombus.^5^ Once the diagnosis is established, treatment includes resolving the current thrombus and preventing further thrombotic events. Anticoagulation with low-molecular-weight heparin as soon as the diagnosis is made has been shown to improve survival.^7^ Transitioning to an oral anticoagulant is then performed. According to the 2016 CHEST guidelines, patients with venous thromboembolism should be treated for a minimum of six months. These guidelines are commensurate with those set forth by the European Association for the Study of the Liver, specifically for mesenteric thrombus.^8^,^9^ After the acute thrombotic event has been stabilized, the patient requires hematologic testing to assess for prothrombotic diseases.^5^

Women using hormonal contraception are approximately four times more likely to develop venous thromboembolism compared to women not prescribed hormonal contraception.^10^ Studies report the association between hormonal contraception and increased risk of thromboembolism is related to the alteration of procoagulant factors and endogenous anticoagulant proteins. The estrogen components of hormonal contraception seem to cause an increase in the procoagulant factors II, VII, VIII, X and fibrinogen, and a decrease in antithrombin and tissue factor pathway inhibitor activity.^11^,^12^ It should be noted that the formulation, administration route, dose, and progesterone type (in combined formulations), all affect the overall risk of thrombosis.^11^,^12^ There is a general misnomer that non-oral forms of hormonal contraception (e.g., etonogestrel/ethinyl estradiol vaginal ring) have a lower risk of thrombotic events compared to oral contraception.

The Transatlantic Active Surveillance on Cardiovascular Safety of NuvaRing study, published in 2013, did not specifically report episodes of mesenteric venous thrombosis, although it did report that episodes of venous and arterial thromboembolism were similar between clinical users of intravaginal contraceptive rings and oral contraceptives.^13^ While mesenteric thrombosis associated with intravaginal contraception is rare, two similar cases have been reported in the literature.^14^,^15^ In addition, when obtaining medication history from patients, non-oral medications can often be missed or discounted by clinicians. Although research is somewhat limited, the etonogestrel/ethinyl estradiol vaginal ring does appear to have the same amount of cardiovascular risk associated with its use as its well-studied counterpart, oral contraceptive pills.^13^

## CONCLUSION

This case highlights that women without known, primary thrombotic risk factors can still suffer major, life-threatening thrombosis when using hormonal contraception, regardless of form. The diagnosis of mesenteric and portal venous thrombosis must be considered in women presenting with abdominal pain who are prescribed hormonal contraceptives, including intravaginal forms.

## Figures and Tables

**Image:**
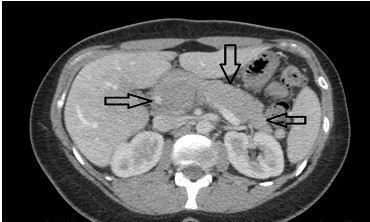
Axial image of abdominal/pelvis computed tomography with contrast demonstrates the thrombus, indicated by the black arrows, in the superior mesenteric vein and extending toward the splenic vein.
